# Unmet Medical Needs and Food Insecurity in Children with Neurodevelopmental Disorders: Findings from the 2019 National Health Interview Survey (NHIS)

**DOI:** 10.3390/children9121798

**Published:** 2022-11-23

**Authors:** Rose Calixte, Elizabeth P. Helzner, Sumaiya Islam, Marlene Camacho-Rivera, Susmita Pati

**Affiliations:** 1 Department of Epidemiology and Biostatistics, SUNY Downstate Health Sciences University, Brooklyn, NY 11203, USA; 2 CUNY School of Medicine, City College of New York, New York, NY 10031, USA; 3 Department of General Public Health, Mailman School of Public Health, Columbia University, New York, NY 10032, USA; 4 Department of Community Health Sciences, SUNY Downstate Health Sciences University, Brooklyn, NY 11203, USA; 5 Department ofPediatrics, Renaissance School of Medicine, Stony Brook, NY 11794, USA; 6 Alan Alda Center for Communicating Science®, Stony Brook University, Stony Brook, NY 11794, USA

**Keywords:** food insecurity, unmet medical needs, neurodevelopmental disorders (NDD)

## Abstract

In the United States, 17% of children ages 3–17 have a developmental disorder. The complexity of care for such children require families to provide a significant amount of health care at home, representing a substantial economic cost. Our study identifies sociodemographic characteristics of children with neurodevelopmental disorders (NDD) that are predictive of unmet medical needs and food insecurity. We modeled the outcomes using a multivariable generalized linear model and a robust Cox proportional hazard model. Among children with NDD, 7.4% reported a delay in obtaining care, 3.6% avoided getting care and 17.3% live in a household that experienced food insecurity. Lack of health insurance and lack of usual source of care increased the risk for cost-related delay in medical care and cost-related avoidance of medical care. Children with NDD whose parents have less than a college degree and those from households with income <$75,000 had increased risk for food insecurity in the past 30 days. Our results underscore the need to implement additional screening to identify children with NDD who are at greater risk for unmet medical and social needs by health care providers and care coordination organizations.

## 1. Introduction

Nearly 20% of children under age 18 in the United States, or about 14.1 million children, have special health care needs, impacting one in four (25%) households with children [1,2]. The U.S. Maternal and Child Health Bureau defines children with special health care needs (CSHCN) as: children with at least one chronic physical, developmental, emotional, or behavioral condition requiring the use of health and related services beyond those required by typical children [3]. Among CSHCN, those with developmental disability, including those with neurodevelopmental disabilities (NDD), represent the largest share [4]. In accordance with the Diagnostic and Statistical Manual of Mental Disorders, 5th Edition (DSM-5), the spectrum of children with neurodevelopmental disorders (NDD) includes attention deficit/hyperactivity disorder (ADHD), intellectual disability (ID), learning disability (LD), developmental disability (DD) and autism spectrum disorder (ASD) [5].

The prevalence of co-occurring conditions among children with special health care needs is highest among children with NDDs compared to neurotypically developed children [4,6]. Various studies have reported greater unmet needs for CSHCN with emotional and behavioral health-related conditions in accessing specialty medical care, speech therapy and mental health services [7,8,9,10]. Furthermore, evidence suggests that the scope of needed services for CSHCN is often not comprehensively delivered in subspecialty locations, leaving parents to be responsible to act as care coordinators [11,12]. In order to offset such burdens, parents of CSHCN spent about 13 h per week providing direct care to the CSHCN or in care coordination [13]. As a result, parents of CSHCN have reported feelings of hopelessness, isolation, frustration, lack of energy and motivation, cognitive difficulties, as well as increased worry regarding their child’s health [14].

Due to the complexity of caring for CSHCN, families in the US provide a significant quantity of health care at home to CSHCN, representing a substantial economic cost. Families who care for the children forego approximately $3200 in earnings every year per child due to missed work [15]. Beyond the economic, physical and mental impact on families of CSHCN, the burden also exists on the educational achievement of CSHCN due to missed school days [16,17].

Among CSHCN, further disparities exist in the burden of unmet needs based on diagnosis [6]. For instance, among children with CSHCN, those with autism spectrum disorder (ASD) have the highest burden of unmet needs [18]. Despite advances in understanding the complexities of care for CSHCN, a recent national study found a significantly higher odds ratio of unmet needs in CSHCN obtaining genetic counseling compared to non-CSHCN [19]. Other cross-sectional studies identified several factors, such as race, parental educational attainment, poverty level and insurance status, which are associated with unmet needs [20,21,22]. It is imperative that we continue to update the research landscape on factors that are most predictive of burdens that are associated with CSHCN and their caregivers to provide appropriate healthcare that includes care coordination delivery models that incorporate adverse social determinants of health [23]. The goal of this study is to identify sociodemographic characteristics of children with neurodevelopmental disorders (NDD) that are predictive of unmet medical needs and food insecurity.

## 2. Materials and Methods

We performed a secondary analysis of the 2019 National Health Interview Survey (NHIS) dataset [24]. These data are nationally representative of the U.S. civilian population as has been described in previous publications [25,26,27,28]. In this study, we restricted the inclusion criteria to children with neurodevelopmental disorders (NDD) as defined in the Diagnostic and Statistical Manual of Mental Disorders, 5th Edition (DSM-5) [5] which represent the largest group of CSHCN [4]. Thus, the analytic sample was restricted to NHIS participants ages 2–17 with any of the following parental-reported diagnosis: attention deficit/hyperactivity disorder, intellectual disability, autism spectrum disorder, other developmental disorder, learning disability and other developmental disability.

We assessed burden of unmet medical needs in NDD relating to medical, dental and prescription care. The first outcome of interest is cost-related delay in care. We define cost-related delay in care by collapsing any yes responses to the question: “During the last 12 months, has the sample child been delayed in getting medical care, filling prescription and dental care because of the cost?” The second outcome of interest is cost-related avoidance of care; we define cost-related avoidance of care by collapsing any “yes” responses to the question: “During the last 12 months, was there any time when sample child needed medical care, filling prescription and dental care but did not get it because of the cost?” We assessed burden of care using food insecurity in the household during the past 30 days. The food security status of the household was determined using the 10-item Food Security Scale, which measured the households’ food situation based on the past 30 days [29]. We dichotomized the 4-level food security variable into those that were food secure versus those that were food insecure.

Predictors of interest were measured both at the child level and at the parental level. Child level predictors were defined to be the following: age, gender, race/ethnicity, US-born (yes/no), insurance status and usual source of care. Responder level predictors included gender of primary responder, age of primary responder, highest parental educational level, legal marital status of caregiver, household income, housing stability and parental employment status.

We measured univariate associations between the outcome variables and the predictors using linear regression for continuous predictor and adjusted Wald chi-square test for categorical predictor, accounting for the complex survey design of the NHIS. All analyses were weighted using the appropriate analytic weight provided in the dataset. We used a multivariable logistic regression model to find independent predictors for unmet medical needs and a robust Cox proportional hazard model to find predictors of food insecurity in NDD due to a higher rate of food insecurity in the study sample [30]. We reported odds ratio (OR) for the logistic regression model and prevalence ratio (PR) for the robust Cox model in cross-sectional studies [31]. All demographic variables were used in the model as primary predictors. Social and economic variables were entered into the multivariable model if statistically significant (i.e., *p*-value set to be ≤ 0.20) in the bivariate analysis using adjusted Wald chi-square. All analyses were conducted using the SAS 9.4^®^ and Stata 16^®^ software programs as appropriate. The significance level was set at 0.05.

## 3. Results

### 3.1. Sample Descriptive

**Figure 1:**
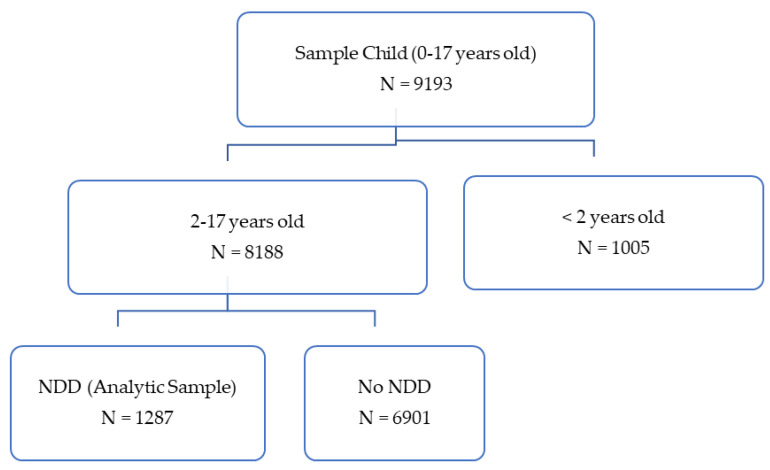
Final Analytic Sample.

The analytic sample included 1287 children between the ages of 2 and 17 with a diagnosis of NDD, representing 14.8% of children (or 9.7 million) in the United States (See Figure 1). The mean age of the analytical sample was 10.7 years (SE ± 0.14 years). The majority (i.e., 66%) of the analytic sample was male. Among children with NDD, 57.5% were non-Hispanic White, 13% were non-Hispanic Black, 17.7% were LatinX and 7.8% were other. Less than 2% of the analytic sample was non-US born. For healthcare status and access outcomes, close to 98% had health insurance, just over 8% reported fair or poor health and 2% reported no usual source of care. Among children with NDD, 7.4% had delayed care in the past 12 months due to cost, 3.6% avoided getting care in the past 12 months due to cost and 17.3% live in a household that experienced food insecurity in the past 30 days.

### 3.2. Univariate and Multivariable Association of Child and Responder Level Predictors with Cost-Related Delay of Care in the Past 12 Months

In Table 1, we present the results of the univariate association of child and parent level predictors with our outcomes of interest. At the child-level, health insurance status and usual source of care were both associated with cost-related delay in medical care (Table 1). At the responder-level, sex of primary responder, education level and home ownership status were associated with cost-related delay in medical care.

After multivariable adjustment (Table 2), compared to US born, foreign born children with NDD had 6.12 times the odds of cost-related delayed medical care (95% CI = [2.25, 16.66]). Additionally, there were higher odds of cost-related delayed medical care in children without health insurance (OR = 4.83, 95% CI = [1.91, 12.21]) compared to children with health insurance and those without a usual source of care (OR = 4.62, 95% CI = [1.46, 14.63]) compared to children with a usual source of care. Responder-level factors significantly associated with cost-related delayed medical care were having a female primary responder (OR = 1.83, 95% CI = [1.06, 3.19]) compared to male, and lack of home ownership (OR = 2.26, 95% CI = [1.25, 4.06]) compared to those who either own their home or are in the process of buying their home.

In models stratified by the sex of the responder, the analysis (Table 3) with race and health insurance of child, age of responders, income and home ownership of caregiver as predictors revealed that, for children with NDD with male as primary responders, younger age was associated with lower odds of cost-related delay in care and lack of health insurance was associated with higher odds of cost-related delay in care. Among children with NDD with female as primary responders, lack of health insurance and those living in houses with non-ownership stake had higher odds of cost-related delay in care.

### 3.3. Univariate and Multivariable Associations of Child and Responder Level Predictors with Cost-Related Avoidance of Care in the Past 12 Months

Cost-related avoidance of care was more common in children with NDD who were uninsured. Responder-level characteristics associated with greater cost-related care avoidance included having a female primary respondent, lower parental education and income, and lack of home ownership (Table 1).

After multivariable adjustment (Table 2), cost-related avoidance of care in the past 12 months was associated with lack of health insurance (OR = 3.5, 95% CI = [1.34, 9.11]), lack of a usual source of care (OR = 3.96, 95% CI = [1.19, 13.15]), moderate parental education (OR = 2.39, 95% CI = [1.26, 4.54] for some college education vs. college degree or more) and mid-level household income (OR = 2.09, 95% CI = [1.04, 4.20] for $50,000 to $74,999 vs. ≥$75,000. Surprisingly, having a high school degree or less was not associated with cost-related avoidance of care vs. those with a college degree or more. Similarly, children with NDD from household with income < $50,000 were not different from the highest income household in avoiding needed care due to cost.

### 3.4. Univariate and Multivariable Child and Responder Level Predictors with Food Insecurity in the Past 30 Days

In the univariate analysis (Table 1), race, age of primary responder, sex of primary responder, highest parental education, income level, parental employment, years in current home and home ownership are all associated with food insecurity.

Education, income and house ownership were significant predictors of food insecurity in the past 30 days (Table 2). Compared to children with NDD from households with at least a college degree, those from households with less than high school degree, high school degree or GED and some college were all at increased risk for food insecurity (PR = 2.57, 95% CI = [1.27, 5.18], PR = 2.04, 95% CI = [1.09, 3.83] and PR = 2.22, 95% CI = [1.20, 4.12], respectively). Additionally, children originating from households with income < $75,000 were shown to be at increased risk for food insecurity with prevalence ratio for those with income < $35,000 being 4.78 (95% CI = [2.32, 9.86]). The prevalence ratio for those with income between $35,000 and $49,999 was 2.95 (95% CI = [1.31, 6.64]), indicating an increase in the risk for food insecurity in the past 30 days compared to those with income ≥ $75,000. For those with income between $50,000 and $74,999, the prevalence ratio for food insecurity was 3.58 (95% CI = [1.66, 7.73]) compared to those with income ≥ $75,000. Lastly, children with NDD living in rented houses or those with other arrangement were at increased risk for food insecurity in the past 30 days (PR = 1.77, 95% CI = [1.20, 2.62]) compared to those living in households who own their homes or are in the process of buying their home.

## 4. Discussion

This study advances our current understanding of the challenges faced by children with NDD and their families. It identifies several child and responder level sociodemographic characteristics that are associated with unmet medical needs and food insecurity in children with NDD. Some of these factors include health insurance status of the child, child having access to usual source of care, sex, income, education level and home ownership status of the primary responder.

Consistent with other studies [32,33,34], we found an overrepresentation of male children with NDD, which may have an X-linked chromosomal effect [35]. However, despite being majorly male, there was no sex differences at the child level in term of access to care and food insecurity.

Considering that the US does not offer universal health insurance, it is worth noting that 98% of children with NDD have access to health insurance. Nonetheless, 7.4% had cost-related delay in medical care in the past 12 months. In our multivariable model, children without health insurance were 4.8 times more likely to have cost-related delay in medical care and 3.5 times more likely to have cost related avoidance of care in the past 12 months. Other studies have also shown that lacking health insurance is associated with unmet medical need [10,16,20].

Based on recent statistics from the 2018–2019 National Survey on Children’s Health, 84.5% of CSHCN did not receive care in a well-functioning system as defined by the Maternal and Child Health Bureau, which comprised six tenets: (1) early and ongoing screening, (2) patient-centered medical home that is coordinated, comprehensive and ongoing, (3) easy access to organized community-based services, (4) access to services necessary to make transitions to adult life, including healthcare, (5) adequate insurance and funding to pay for services they need and (6) families as partners in decision-making at all levels of care, from direct care to the organizations that serve them [12,36]. So, in addition to the unmet medical needs related to lacking a usual source of care, the vast majority of CSHCN are not receiving adequate care [36]. Furthermore, studies on CSHCN have shown a positive effect of health insurance on healthcare access and utilization. Current literature also delineates that higher family income and insurance coverage have been linked to an increased likelihood of obtaining specialty healthcare services for CHSCN [37,38].

Children with NDD who were foreign-born were shown to be at an increased risk of having cost-related delay in medical care compared to those who were US-born. Other studies using NHIS survey data have shown that children born outside of the US, particularly in Mexico, Central America, South America, Asia and the Indian subcontinent, were at higher risk of being uninsured than US-born children. Furthermore, US children born in South America were at higher risk of cost-related delay in care and cost related forgone care compared to US born children [39]. Children who are immigrants are also less likely to access care than non-immigrants [40,41,42]. Some of the potential contributing factors may be immigration status, language barrier and job insecurity.

In our sample, 70% of adult respondents of children with NDD were female and NDD children with female respondents were 1.83 times more likely to have a cost-related delay in medical care. Previous studies have similarly found a high ratio of female caregivers for CSHCN 15. This highlights the longstanding and persistent inequality in the gender distribution of caregiving roles [43], wherein women are more likely than men to have greater childcare responsibilities, yet have less access to family related employment leave benefits [44,45]. When we stratified our analysis by respondent sex, we found sex-related differences in characteristics associated with cost-related delayed care. While health insurance status was an important predictor in both groups, age of respondent was important among male respondents only, with male responders in the younger than 55 age group being less likely to report cost-related delay in care for the child, and home ownership was important among female respondents only. These results highlight the need to have multifaceted programs in place to address different needs of caregivers of children with NDD. However, small cell sizes precluded robust estimates in these sex-stratified models.

Another primary outcome that we examined in our study was food insecurity. We found that 17.3% of children with NDD lived in households that were food insecure, with significant associations found with lower parental income, lower parental education level and lack of home ownership (all indicators of lower SES overall). This finding is consistent with the fact that food insecurity is more than twice as common in low-income households [46]. Food insecurity itself has been linked with many other adverse outcomes in children, including increased risk of asthma and depressive symptoms [47], iron deficiency, lower school-based cognitive performance and higher rates of tooth decay [48,49,50,51]. Food insecurity in children has also been linked to avoidance of health care due to costs, as well as higher rates of emergency department visits [47]. Given that food insecurity is associated with higher rates of adverse child health outcomes and children with NDD are particularly vulnerable, specialized policy approaches should be considered to address food insecurity in this special population.

## 5. Strengths and Limitations

There are several strengths to this study. Since NHIS is a nationally representative survey, these results can be generalized to all US children aged 2–17 with NDD. Our models included both child- and caregiver-level socioeconomic characteristics that may be associated with unmet medical need and food insecurity. The use of food insecurity assessment based on the past 30 days reduced the likelihood of recall bias compared to studies that used a longer recall period. Additionally, for food insecurity, we used a robust Cox proportional hazard analysis to address issues related to logistic model of non-rare outcomes.

Nevertheless, the study is limited by the cross-sectional nature of the data, which precluded our ability to infer causal relationships between sociodemographic factors and our healthcare-related outcomes. Since delayed and avoided medical care was ascertained for the past 12 months, some recall bias may be introduced in the measurement of these outcomes, though an argument could be made that unmet medical needs due to cost may not be impacted by recall bias. Additionally, the survey instrument did not indicate if the parent respondent is the primary caregiver. Lastly, the size of the data prevented us from disaggregating the model further by type of disability without affecting model fit. Future studies should look into pooled NHIS data to increase the size of each condition to further develop models of unmet need for each condition.

## 6. Conclusions

Our study presents many avenues of opportunity for interventions to mitigate the negative impact of unmet medical needs and food insecurity for CSHCN, who are underserved and are vastly disadvantaged. In summary, at the child level place of birth, health insurance status and access to usual source of care were associated with unmet medical needs. At the responder level, income, education and home ownership status were associated with food insecurity. To alleviate the burden and provide more equitable access for this vulnerable population, screening and care coordination programs can be implemented at point of care to direct children with NDD and their caregivers to community services.

## Figures and Tables

**Table 1:** Univariate Analysis of Unmet Medical Needs and Food Insecurity with Social demographic Characteristics.

		Cost-Related Delay in Care in the Past 12 Months			Cost-Related Avoidance of Care in the Past 12 Months			Food Insecurity in the Past 30 Days		
	Full Sample	Yes	No	*p*-Value	Yes	No	*p*-Value	Yes	No	*p*-Value
Child Level Predictors										
Age (mean (SE))	10.7 (0.14)	11.1 (0.46)	10.6 (0.14)	0.285	10.6 (0.15)	11.3 (0.41)	0.141	10.7 (0.15)	10.6 (0.38)	0.825
Sex				0.615			0.314			0.963
Male	66.4 (1.60)	69.0 (5.59)	66.2 (1.63)		60.8 (5.61)	66.7 (1.64)		66.4 (3.89)	66.2 (1.78)	
Female	33.6 (1.60)	31.0 (5.59)	33.8 (1.63)		39.2 (5.61)	33.3 (1.64)		33.6 (3.89)	33.8 (1.78)	
Race				0.179			0.115			<0.001
Non-Hispanic White	57.5 (1.80)	45.5 (5.91)	58.4 (1.82)		42.3 (6.24)	58.6 (1.84)		37.5 (4.45)	62.5 (1.86)	
Non-Hispanic Black	13.0 (1.34)	14.4 (3.86)	12.9 (1.38)		19.5 (6.57)	12.5 (1.34)		20.5 (3.99)	11.4 (1.35)	
Hispanic	21.7 (1.62)	33.5 (5.44)	20.81 (1.62)		29.9 (5.32)	21.1 (1.64)		34.8 (4.91)	19.2 (1.63)	
Non-Hispanic Other	7.8 (0.93)	6.6 (2.70)	7.9 (0.97)		8.3 (5.32)	7.73 (0.96)		7.2 (2.07)	7.9 (0.99)	
US Born				0.077			0.259			0.605
Yes	98.3 (0.44)	93.2 (2.83)	98.7 (0.41)		95.6 (2.44)	98.5 (0.43)		97.8 (1.03)	98.4 (0.45)	
No	1.7 (0.44)	6.82 (2.83)	1.3 (0.41)		4.4 (2.44)	1.5 (0.43)		2.2 (1.03)	1.6 (0.45)	
Health Insurance				0.017			0.043			0.280
Yes	98.2 (0.38)	91.7 (2.94)	98.72 (0.32)		93.1 (2.75)	98.6 (0.33)		97.0 (1.35)	98.4 (0.33)	
No	1.8 (0.38)	8.3 (2.94)	1.28 (0.32)		6.9 (2.75)	1.4 (0.33)		3.0 (1.35)	1.6 (0.33)	
Usual Source of Care				0.045			0.059			0.078
Yes	97.3 (0.59)	90.0 (3.79)	97.9 (0.57)		90.7 (3.71)	97.8 (0.57)		94.2 (1.94)	97.9 (0.58)	
No	2.7 (0.59)	10.0 (3.79)	2.1 (0.57)		9.3 (3.71)	2.17 (0.57)		5.8 (1.94)	2.1 (0.58)	
Responder Level Predictors										
Age of Primary Responders				0.479			0.505			0.020
20–34	28.7 (1.54)	36.2 (5.92)	28.1 (1.61)		33.5 (5.48)	28.4 (1.62)		33.0 (3.91)	27.7 (1.67)	
35–44	43.9 (1.66)	36.8 (5.20)	44.5 (1.72)		41.1 (4.97)	44.0 (1.73)		46.3 (4.00)	43.4 (1.80)	
45–54	21.1 (1.18)	19.5 (4.07)	21.2 (1.24)		16.9 (3.53)	21.5 (1.26)		13.2 (2.55)	22.9 (1.32)	
≥55	6.3 (0.72)	7.5 (3.04)	6.2 (0.74)		8.4 (2.85)	6.1 (0.74)		7.5 (2.18)	6.1 (0.75)	
Sex of Primary Responders				0.012			0.049			<0.001
Male	30.0 (1.70)	19.2 (4.05)	30.8 (1.79)		20.8 (4.37)	30.7 (1.79)		18.2 (3.59)	32.5 (1.85)	
Female	70.0 (1.70)	80.8 (4.05)	69.2 (1.79)		79.2 (4.37)	69.3 (1.79)		81.2 (3.59)	67.5 (1.85)	
Highest Parental Education Level				0.017			0.002			<0.001
<High School	8.2 (1.08)	9.9 (3.60)	8.1 (1.12)		11.1 (3.98)	8.0 (1.13)		17.4 (3.83)	6.4 (1.00)	
High School Graduate or GED	21.5 (1.45)	15.1 (3.81)	22.0 (1.55)		22.5 (5.00)	21.4 (1.53)		30.8 (4.03)	19.5 (1.55)	
Some College	32.0 (1.55)	47.8 (5.51)	30.7 (1.62)		47.3 (5.23)	30.8 (1.63)		42.1 (4.13)	30.0 (1.65)	
College Degree or More	38.3 (1.68)	27.1 (5.10)	39.2 (1.75)		19.0 (4.58)	39.8 (1.73)		9.7 (2.43)	44.2 (1.86)	
Income				0.097			0.009			<0.001
<$35,000	29.6 (1.63)	32.3 (5.46)	29.3 (1.69)		34.2 (7.51)	29.1 (1.69)		61.9 (4.33)	22.9 (1.59)	
$35,000-$49,999	12.1 (1.11)	12.5 (3.08)	12.0 (1.19)		18.4 (6.32)	11.6 (1.11)		12.9 (3.33)	12.0 (1.21)	
$50,000-$74,999	16.7 (1.30)	25.9 (5.04)	16.0 (1.34)		24.4 (5.18)	16.0 (1.35)		17.7 (3.62)	16.5 (1.41)	
≥$75,000	41.7 (1.73)	29.3 (5.31)	42.7 (1.78)		23.0 (5.10)	43.16 (1.74)		7.4 (2.13)	48.6 (1.87)	
Parental Employment Status				0.890			0.878			0.014
At Least one Parent is Working	83.1 (1.25)	83.7 (4.59)	80.0 (1.31)		82.4 (4.83)	82.2 (1.32)		75.3 (3.59)	84.8 (1.30)	
Parents currently unemployed	16.9 (1.25)	16.3 (4.59)	17.0 (1.31)		17.6 (4.83)	16.8 (1.32)		24.7 (3.59)	15.2 (1.30)	
Years in Current Home				0.788			0.918			0.001
0–3 years	47.6 (1.79)	48.0 (5.53)	47.6 (1.84)		49.7 (5.12)	47.4 (1.87)		61.7 (4.19)	44.7 (1.91)	
4–10 years	37.7 (1.75)	39.6 (5.03)	37.5 (1.80)		36.0 (5.51)	37.8 (1.80)		29.5 (4.15)	39.6 (1.86)	
More than 10 years	14.7 (1.09)	12.4 (3.58)	14.9 (1.15)		14.3 (3.79)	14.7 (1.13)		8.8 (2.32)	15.8 (1.20)	
House Ownership				0.002			0.007			<0.001
Own or Being Bought	58.3 (1.74)	39.22 (5.58)	59.8 (1.80)		41.3 (5.80)	59.7 (1.78)		27.4 (3.95)	64.7 (1.84)	
Rented and Other Arrangement	41.7 (1.74)	60.77 (5.58)	40.2 (1.80)		58.73 (5.80)	40.3 (1.78)		72.6 (3.95)	35.3 (1.84)	

Data are summarized using weighted mean and standard error (SE), weighted percent (SE). Univariate association of sociodemographic characteristics with unmet medical needs and food insecurity were measured using adjusted Wald chi-square test and linear regression with categorical predictors. Standard errors were computed using Taylor-series linearization. Results with *p*-value < 0.05 are considered significant. Variables with *p*-value < 0.2 are then entered in the multivariable model.

**Table 2:** Sociodemographic correlates of unmet medical needs and food insecurity in children with NDD.

	Cost-Related Delay in Care in the Past 12 Months		Cost-Related Avoidance of Care in the Past 12 Months		Food Insecurity in the Past 30 Days	
	OR	95% CI	OR	95% CI	PR	95% CI
Child Level Predictors						
Age (1-year increase)	1.07	1.00, 1.14	1.06	0.99, 1.13	1.00	0.96, 1.05
Sex						
Male	1.18	0.68, 2.04	0.78	0.47, 1.30	0.96	0.72, 1.29
Female	1.00	1.00, 1.00	1.00	1.00, 1.00	1.00	1.00, 1.00
Race						
Non-Hispanic White	1.00	1.00, 1.00	1.00	1.00, 1.00	1.00	1.00, 1.00
Non-Hispanic Black	1.09	0.52, 2.28	1.60	0.65, 3.93	1.16	0.77, 1.75
Hispanic	1.80	0.99, 3.28	1.57	0.79, 2.10	1.25	0.87, 1.80
Non-Hispanic Other	0.88	0.30, 2.59	1.35	0.49, 3.74	1.15	0.69, 1.90
US Born			-	-	-	-
Yes	1.00	1.00, 1.00	1.00	1.00, 1.00	1.00	1.00, 1.00
No	6.12	2.25, 16.66 ***	2.66	0.67, 10.57	1.48	0.75, 2.90
Health Insurance					-	-
Yes	1.00	1.00, 1.00	1.00	1.00, 1.00	1.00	1.00, 1.00
No	4.83	1.91, 12.21 ***	3.50	1.34, 9.11 *	1.10	0.53, 2.32
Usual Source of Care						
Yes	1.00	1.00, 1.00	1.00	1.00, 1.00	1.00	1.00, 1.00
No	4.62	1.46, 14.63 **	3.96	1.19, 13.15 *	1.52	0.96, 2.41
Responder Level Predictors						
Age of Primary Responder						
20–34	1.38	0.45, 4.21	0.91	0.35, 2.39	0.57	0.30, 1.06
35–44	0.74	0.28, 1.95	0.68	0.30, 1.55	0.66	0.38, 1.16
45–54	0.93	0.34, 2.56	0.71	0.29, 1.75	0.58	0.32, 1.05
≥55	1.00	1.00, 1.00	1.00	1.00, 1.00	1.00	1.00, 1.00
Sex of Primary Responder						
Male	1.00	1.00, 1.00	1.00	1.00, 1.00	1.00	1.00, 1.00
Female	1.83	1.06, 3.19 *	1.51	0.84, 2.72	1.31	0.88, 1.96
Highest Parental Education Level						
<High School	0.90	0.33, 2.51	1.88	0.64, 5.55	2.57	1.27, 5.18 **
High School Graduate or GED	0.60	0.29, 1.26	1.45	0.65, 3.26	2.04	1.09, 3.83 *
Some College	1.60	0.85, 3.01	2.39	1.26, 4.54 **	2.22	1.20, 4.12 *
College Degree or More	1.00	1.00, 1.00	1.00	1.00, 1.00	1.00	1.00, 1.00
Income	-	-	-	-	-	-
<$35,000	0.74	0.33, 1.69	0.99	0.45, 2.19	4.78	2.32, 9.86 ***
$35,000-–$49,999	0.81	0.35, 1.84	1.55	0.60, 4.01	2.94	1.31, 6.64 **
$50,000–$74,999	1.86	0.93, 3.74	2.09	1.04, 4.20 *	3.58	1.66, 7.73 ***
≥$75,000	1.00	1.00, 1.00	1.00	1.00, 1.00	1.00	1.00, 1.00
Parental Employment Status	-	-	-	-		
At Least one Parent is Employed	1.00	1.00, 1.00	1.00	1.00, 1.00	1.00	1.00, 1.00
Parents currently unemployed	0.88	0.42, 1.86	0.79	0.37, 1.71	0.87	0.62, 1.22
Years in Current Home	-	-	-	-		
0–3 years	-	-	-	-	1.14	0.67, 1.93
4–10 years	-	-	-	-	0.88	0.48, 1.60
More than 10 years	-	-	-	-	1.00	1.00, 1.00
House Ownership						
Own or Being Bought	1.00	1.00, 1.00	1.00	1.00, 1.00	1.00	1.00, 1.00
Rented and Other Arrangement	2.26	1.25, 4.06 **	1.51	0.87, 2.63	1.77	1.20, 2.62 **

The outcome variables were analyzed using multivariable logistic regression models and robust proportional hazard regression model. Standard errors were computed using Taylor-series linearization. Data are summarized using odds ratio (OR) and prevalence ratio (PR) with 95% confidence Interval (CI). * *p* < 0.05, ** *p* < 0.01, *** *p* < 0.001.

**Table 3:** Sex-stratified multivariable model of cost-related delay in medical care.

	Cost-Related Delay in Care in the Past 12 Months Among Male Responders		Cost-Related Delay in Care in the Past 12 Months Among Female Responders	
	OR	95% CI	OR	95% CI
Child Level Predictors				
Race				
Non-Hispanic White	1.00	1.00, 1.00	1.00	1.00, 1.00
Non-Hispanic Black	0.46	0.05, 4.04	1.24	0.53, 2.95
Hispanic	1.46	0.50, 4.30	1.57	0.80, 3.07
Non-Hispanic Other	0.58	0.07, 5.14	1.02	0.34, 3.03
Health Insurance				
Yes	1.00	1.00, 1.00	1.00	1.00, 1.00
No	7.84	1.47, 41.81 *	6.79	2.18, 21.19 ***
Parental Level Predictors				
Age of Primary Caregiver				
20–34	0.07	0.01, 0.47 **	4.70	0.88, 25.08
35–44	0.16	0.04, 0.56 **	2.93	0.56, 15.28
45–54	0.20	0.05, 0.75 **	3.98	0.73, 21.67
≥55	1.00	1.00, 1.00	1.00	1.00, 1.00
Income				
<$35,000	2.04	0.53, 7.79	0.59	0.26, 1.33
$35,000–$49,999	0.46	0.05, 3.97	0.86	0.36, 2.07
$50,000–$74,999	1.89	0.47, 7.68	2.04	0.91, 4.60
≥$75,000	1.00	1.00, 1.00	1.00	1.00, 1.00
House Ownership				
Own or Being Bought	1.00	1.00, 1.00	1.00	1.00, 1.00
Rented and Other Arrangement	1.42	0.69, 7.75	2.52	1.31, 4.86 **

The gender-stratified analysis for cost-related delay in care was carried out using multivariable logistic regression model using domain analysis to maintain the complex survey design methodology. Standard errors were computed using Taylor-series linearization. Data are summarized using odds ratio (OR). * *p* < 0.05, ** *p* < 0.01, *** *p* < 0.001.

## Data Availability

The data used in this study can be downloaded from https://www.cdc.gov/nchs/nhis/2019nhis.htm (accessed on 2 February 2021).
